# Expanding provision of professional education for nurses, Africa

**DOI:** 10.2471/BLT.23.289628

**Published:** 2023-03-01

**Authors:** Edgar Manuel Cambaza

**Affiliations:** a ISCED Open University (UnISCED), Rua Carlos Pereira, parcela nº 148/07, Estoril Expansão, 0504-01 Beira, Sofala, Mozambique.

Shortage of nurses harms patient outcomes and health care. In sub-Saharan Africa, insufficient resources and lack of trained educators have limited nursing education, leading to shortages. Online nursing education, particularly blended learning, could help rural and underserved areas without universities to increase their number of nurses, by providing cost–effective quality education. Students would not have to spend on transportation, books and other costs as much as in the traditional face-to-face learning model. Blended learning, a combination of remote learning and clinical components, is a potential answer to the shortage of nurses in sub-Saharan Africa.^1^^,^^2^

Blended learning potentially increases access to education for aspiring nurses who may not have the opportunity to attend traditional, on-campus programmes.^3^ Many individuals in sub-Saharan Africa face barriers to higher education such as financial constraints, geographic isolation and family responsibilities.^4^ Blended learning removes these barriers by providing flexible and affordable learning options accessible from anywhere with an internet connection.

Blended learning can also address the shortage of nursing educators in sub-Saharan Africa. On-campus programmes require many qualified educators, but many countries in the region do not have enough qualified nursing educators.^4^ Educators from other countries can deliver blended learning programmes using technologies such as video conferencing, helping to overcome this shortage.

Blended learning can also improve the quality of nursing education in sub-Saharan Africa by incorporating the latest research and best practices in nursing education. Instructors can regularly update such learning to ensure students receive current and relevant information.^5^ Blended learning programmes provide students with interactive learning experiences, such as virtual clinical simulations and online discussions with peers and educators.

Last, blended learning can contribute to the professional development of nurses in sub-Saharan Africa. These programmes offer continuing education and professional development opportunities for practising nurses, allowing them to stay current with the latest field developments and to improve their skills and knowledge.^6^

Online nursing education is growing in popularity due to its cost–effectiveness and scalability. The lack of (or reduced) housing and transportation costs,^7^ and the ability to accommodate more students make online education more affordable.^8^ Increased internet and technology access in Africa has made online education a viable option for those lacking access to traditional nursing education.^9^

Despite these advantages, blended learning in nursing faces several challenges and limitations such as reliable internet access, low computer literacy among students and instructors, reduced face-to-face interactions, supervision and mentorship, and hands-on clinical experience,^3^^,^^10^ affecting students’ learning experience and support.^11^ The effectiveness of blended learning in nursing may vary depending on the student’s learning style and motivation.^1^ Some students may prefer the flexibility and self-paced nature of blended learning, while others may find it more challenging to stay motivated and focused without the structure and support of an on-campus programme. Therefore, this type of learning alone may not address the nursing shortage in sub-Saharan Africa.

To contribute to addressing the shortage of qualified nurses in sub-Saharan Africa, blended learning must incorporate effective implementation strategies such as clinical placements and support for computer literacy.^12^ Virtual office hours and discussion forums can reduce face-to-face interaction, and clinical placements or simulations can ensure hands-on clinical experience. Clear learning objectives, regular assessments and support services can improve student success.

Policy-makers can create favourable regulations and policies supporting online nursing education’s growth. Such regulations include providing financial incentives for nursing education, improving working conditions for nurses, and addressing the shortage’s root causes, such as inadequate infrastructure and lack of political commitment.^10^ In addition, ensuring the quality and credibility of blended learning programmes through accreditation by reputable bodies, adherence to best practices in distance education, and regular evaluations of the programme’s effectiveness is essential.

In conclusion, open-distance higher education can extend nursing education and address sub-Saharan Africa’s nursing deficit. Blended learning could enhance nursing education, promote access, address the nursing educator shortage and help nurses develop professionally. To maximize the benefits of blended learning in nursing, universities and governments must address these difficulties and support students through guidance, technical support or scholarships. Blended learning can improve health-care outcomes and reduce the regional shortage of nurses through the best practices and student assistance. 
